# “Same Same or Adapted?” Therapists’ Feedback on the Implementation of Trauma-Focused Cognitive Behavioral Therapy With Unaccompanied Young Refugees

**DOI:** 10.32872/cpe.5431

**Published:** 2021-11-23

**Authors:** Johanna Unterhitzenberger, Sophia Haberstumpf, Rita Rosner, Elisa Pfeiffer

**Affiliations:** 1 Department of Psychology, Catholic University Eichstätt-Ingolstadt, Eichstätt, Germany; 2 Center for Mental Health, Department of Psychiatry, Psychosomatics and Psychotherapy, University Hospital Würzburg, Würzburg, Germany; 3 Clinic for Child and Adolescent Psychiatry/Psychotherapy, Ulm University, Ulm, Germany; Philipps-University of Marburg, Marburg, Germany

**Keywords:** TF-CBT, cultural adaptation, refugee, therapist, adolescent

## Abstract

**Background:**

Rates of trauma exposure and posttraumatic stress disorder (PTSD) are high among refugee youth. Although there is a vast evidence base on effective trauma-focused interventions for children and adolescents, there is only limited understanding of how to adapt these interventions for oftentimes severely traumatized young refugees. This study aims to investigate adaptations undertaken during trauma-focused cognitive behavioral therapy (TF-CBT) in a pilot study with unaccompanied refugee minors (URMs).

**Method:**

Written answers on five questions given by N = 9 therapists on N = 16 TF-CBT cases were analysed qualitatively using Mayring’s content analysis. The questions were on (1) additional techniques used in the sessions, (2) obstacles to TF-CBT treatment, (3) cultural factors considered and most helpful components for (4) patient and (5) therapist. The categories were built inductively and analysed descriptively.

**Results:**

In addition to the regular TF-CBT components, added content mostly concerned the so-called “crisis of the week”, meaning a more lengthy discussion of struggles and concerns in their daily lives. Few obstacles in treatment were reported, and little cultural factors had to be considered. The implementation of a trauma narrative and the agenda provided by the manual were frequently reported as helpful.

**Conclusion:**

The results of this study indicate that the manualized evidence-based treatment TF-CBT can be used in the culturally heterogeneous population of URMs with minor adaptations. These findings can contribute to future research as well as clinical practice with URMs.

## Highlights

- TF-CBT is a promising treatment for PTSD in traumatized refugee minors.
- Necessary adaptations for this target group have not been analysed so far.
- Therapists reported only a few “on the fly” adaptations during a pilot study on TF-CBT.

Unaccompanied refugee minors (URMs) constitute a vulnerable population, firstly due to their various traumatic experiences before, during and after their flight (Reed et al., 2012; Steel et al., 2017), and secondly in terms of severe post-migration stressors (Keles et al., 2018) on their arrival in the host country. It comes as no surprise that the prevalence rates of trauma- and stress-related mental health conditions are higher among URMs compared to youth without a migration background, immigrant samples (Betancourt et al., 2017) or accompanied refugee minors (Bean et al., 2007). A recent meta-analysis on mental illness among refugee minors revealed that 23% report posttraumatic stress disorder (PTSD) (Blackmore et al., 2020).

Current treatment guidelines for treating trauma-related disorders, especially PTSD, recommend trauma-focused cognitive behavioral approaches (International Society for Traumatic Stress Studies [ISTSS], 2019; Rosner et al., 2019) for traumatized children and adolescents. In this context, trauma-focused cognitive behavioral therapy (TF-CBT, e.g. the specific manual by Cohen et al., 2017) has been identified as a gold standard treatment for children and adolescents with PTSD across guidelines and meta-analyses (Gutermann et al., 2016). Experts claim, however, that yet child trauma guidelines focus too little on children’s cultural background and possible adaptations (Alisic et al., 2020).

Most evidence-based treatments for PTSD were developed in western societies. They were then increasingly widely implemented and found to be effective in samples with cultures outside of western societies (Ennis et al., 2020). Only a very small number of interventions have been specifically developed and tailored to the needs of URMs, for example the trauma-focused group intervention “Mein Weg” (English “My Way”) (Pfeiffer et al., 2018), which is based on TF-CBT but re-modeled into a group-based low-level intervention for refugees in child welfare programs. The development of this intervention adopted a theory-driven approach for cultural adaptation (Heim & Kohrt, 2019). The theory-driven changes focused mostly on delivery in a group format (e.g. additional group discussions), the language barrier (e.g. changes in materials) and the inclusion of flight and migration specific content (flight route as part of narrative). More commonly, evidence-based trauma-focused treatments are adapted to cultural characteristics of study populations in a data-driven, so-called “bottom-up” approach. These approaches are especially favorable if the question is whether there is a good fit between the evidence-based treatment itself and the new target group, and whether cultural adaptations are necessary to some or all aspects of that specific therapy.

A recent review (Ennis et al., 2020) systematically reviewed research articles on cultural adaptations in trauma-focused CBT approaches with children and adults. The results highlight the complexity of cross-cultural adaptations of psychotherapy due to several reasons such as heterogeneous sources of information (e.g. stakeholders, therapists or patients), the usage of different frameworks for cultural adaptations (if used at all) and different levels of efficacy evaluation. Seven out of the 17 included studies were on cultural adaptations in TF-CBT. They either implemented the treatment with immigrant samples in western countries (e.g. Schottelkorb et al., 2012) or delivered the treatment abroad in the cultural context of the country itself (DRC: McMullen et al., 2013; Jordan: Damra et al., 2014; Zambia: Murray et al., 2013; Tanzania: O’Donnell et al., 2014). One study involved local therapists (Murray et al., 2013) as main source of information for assessing potentially necessary changes to the treatment protocol during treatment delivery, while others used focus groups, surveys, or expert panels ahead of treatment implementation. The most often used source in the studies described in the review by Ennis et al. (2020), which focused on data-driven approaches, were (local) therapists as they might function as a direct mediator between high adherence in the implementation of the manual on the one hand and the individual needs of their patients on the other hand.

There are only two studies on cultural adaptations to the TF-CBT protocol delivered to refugee minors (Schottelkorb et al., 2012; Unterhitzenberger et al., 2019). The refugee population might throw up specific challenges as it represents a heterogeneous population that originates from different countries and cultures. Consequently, this makes an oftentimes preferred “one size fits all” approach even more challenging. The authors of both studies gave only a brief description of marginal changes to the protocol (e.g. translation when needed, tailored psychoeducation, more sessions on trauma narrative). This leaves a gap in the literature on the need for cultural adaptations in TF-CBT for this vulnerable cohort. Especially so-called “on the fly” adaptations of experienced therapists (Heim & Kohrt, 2019) might be crucial to increasing understanding of necessary adaptations to evidence-based trauma-focused treatments such as TF-CBT for URMs.

In our recent pilot study (Unterhitzenberger et al., 2019) on TF-CBT with URMs, we implemented the TF-CBT protocol without prior theory-driven adaptations. The aim was to evaluate the feasibility of TF-CBT for this specific target group. Therapists were instructed to provide the treatment according to the manual, however, also to implement and document any “on the fly” adaptations they made in order to successfully treat the refugee patient. The present study, which was part this pilot study (Unterhitzenberger et al., 2019), aims to increase knowledge on how to adapt TF-CBT to the specific needs of URMs by examining therapists’ self-reported cultural adaptations in implementing TF-CBT with URMs in a qualitative study design.

## Method

This study is part of a recently published pilot study conducted in Germany between March 2015 and July 2017. For details on the procedure please refer to the main publication (Unterhitzenberger et al., 2019). The participants treated in this pilot study were 26 male URMs (*M_age_* = 17.1; *SD* = 1.0; range 15-19) who had been in Germany for an average of 9.8 months (*SD* = 3.9) and who originated from eight different countries in the Middle East and Africa. 22 of them completed treatment. Uncontrolled effect sizes were high for PTSD symptoms at post (*d* = 1.08) and follow-up assessments (*d* = 1.23).

### Participants

A total of 9 therapists were taken into account for this analysis. Please see Table 1 for the description of participants’ characteristics. Therapists responded to the survey for a total of 16 treatment cases. Unfortunately, we do not have therapist feedback on all the cases treated, as the questionnaire was put together during the ongoing pilot study. All therapists participated in a TF-CBT training by a certified trainer and received biweekly supervision. In addition, one of the manual developers offered case consultation calls once a month. During the project, an expert in psychotherapy with refugees and torture survivors ran a half-day training session attended by all therapists.

**Table 1:** Sociodemographic Characteristics of the Participating Therapists (N = 9)

Characteristic	
Age *M* (*SD*)	35 (9.5)
range	29 – 60
Gender *n* (%)	
female	8 (88.9)
CBT therapist *n* (%)	9 (100)
Licensed	6 (66.7)
In training	3 (33.3)
Child and adolescent therapist	1 (11.1)
Adult therapist with additional training for children	8 (88.9)
Clinical experience *n* (%)	
1 to 5 treated cases	2 (25.0)
11 to 20 treated cases	1 (12.5)
21 to 50 treated cases	1 (12.5)
> 50 treated cases	4 (50.0)
TF-CBT cases treated *M* (*SD*)	5.5 (2.99)
range	0 – 20

*Note*. CBT = cognitive behavioral therapy; TF-CBT = trauma-focused cognitive behavioral therapy; *M* = mean; *SD* = standard deviation.

### Intervention

The TF-CBT treatment protocol followed the manual by Cohen et al. (2017). It consists of nine treatment modules on psychoeducation and parenting skills, relaxation, affective modulation, cognitive processing, trauma narrative and cognitive processing II, in vivo exposure, conjoint child/caregiver session, and enhancing safety and future skills. Standard TF-CBT involves twelve 90-minute sessions with the child and the caregiver. Usually, the caregiver is a parent, however, for children and adolescents housed in child welfare facilities (like URMs) these are professionals, for instance, social workers. The amount of caregiver involvement depends on the child’s age. According to the manual developers, TF-CBT is flexible and culturally sensitive (Cohen et al., 2017). In this pilot study, treatment fidelity was relatively high (62-82%) (Unterhitzenberger et al., 2019). The mean treatment dose was 15 sessions. An interpreter was present in 55% of treatment cases.

### Data Collection

After each session, therapists filled out a session checklist for the TF-CBT module addressed in the respective session to report on treatment adherence. After the responses to the components (yes/no), one item “additional content or techniques” was to be answered openly that was analyzed for this article (Question 1). Furthermore, we conducted a survey among therapists. They were given a questionnaire for each study case at the same day they had completed it. The questionnaire consisted of four Likert-scaled questions and eleven questions on each treatment case. It included questions on the complexity of the disorder, therapeutic relationship, therapist’s satisfaction, helpful components and obstacles as well as cultural considerations. For the purpose of this study, we present responses from the following four questions that we deemed to be helpful regarding (cultural) adaptations: “Which component(s) constituted an obstacle in treatment?” (Question 2), “Did you consider cultural factors in this treatment case? If so, which ones?” (Question 3); “Which TF-CBT component(s) helped the patient most?” (Question 4); “Which TF-CBT component(s) was/were especially helpful for you in treatment?” (Question 5).

### Data Analysis

The answers were analyzed according to Mayring’s qualitative content analysis (Mayring, 2000). We conducted categories in a structured manner. A key component of this process is the coding manual (for an overview see Supplementary Material). The coding manual is developed in three steps: the definition of categories, the derivation of examples from the text, and the addition of rules for coding when necessary. Categories were built inductively, meaning they were derived from the material rather than from a theoretical concept. The coding was done by SH, any uncertainties regarding the coding and coding manual were discussed with JU. The categories were then analyzed quantitatively by percentage of naming. This approach seemed suitable, as many answers were very short or only bullet points. Categories were built separately for each question so there is a coding manual for each question. For Question 1, 242 session checklists were analyzed. For Questions 2 to 5, we analyzed 16 questionnaires from 16 treatment cases. Percentages represent how often one category was named by the answers analyzed for the respective question. Percentages represent data from one question and all categories for the respective question are reported except for Question 1, where we report only categories with percentages ≥ 4.

## Results

### Additional Content or Techniques

In 150 out of the 242 checklists, the therapists named 172 additional contents or techniques. We coded them in 21 subcategories. The categories named most often were “crisis of the week” (12.2%), psychoeducation (11.3%), cognitive processing and trauma narrative (each 11.1%). These were followed by relaxation (4.7%), treatment course, grief, and affective modulation (4.1% respectively).

### Obstacles in Treatment

Five therapists named obstacles regarding TF-CBT for six cases. Eleven responses categorized in six categories showed the following challenges in implementing TF-CBT: relaxation (36.4%), cognitive processing I and TF-CBT components ahead of trauma narrative (each 18.2%), affective modulation, work sheets and linguistic problems (each 9.1%).

### Cultural Factors in Treatment

Nine therapists gave 14 responses that indicate consideration of cultural factors that were assigned to eleven categories: using pride, religion, and metaphors (each 14.3%), simplify language, using strength and respect, culture-specific grief rituals, combination of psychological and somatic complaints, information on the culture-specific image of women, handling of aggressive behavior, culture-specific adaptation of treatment relationship and handling of general cultural controversies (each 7.1%). For six cases, therapists did not describe any cultural considerations. Three of the participants indicated that they possibly did but were not aware of it or did not explicitly do so.

### Most Helpful for Patient

Twenty-six responses for 15 treatment cases were given regarding the most helpful TF-CBT components for the patient: trauma narrative (53.9%), cognitive processing (19.2%), psychoeducation, relaxation, conjoint session with patient and caregiver (each 7.7%) and affective modulation (3.9%).

### Most Helpful for Therapist

Eight therapists responded to the question about what was most helpful for their work derived from TF-CBT in 22 responses for 13 treatment cases: having an agenda (22.7%), trauma narrative and psychoeducation (each 18.2%), cognitive processing (13.6%), intervision with other TF-CBT therapists (9.1%) and affective modulation, conjoint session, grief modules, and expectation of treatment success (each 4.6%).

## Discussion

This is the first study to investigate “on the fly” adaptations by practitioners implementing TF-CBT with an especially vulnerable and diverse population. As one of the first studies, we present qualitative findings from therapists’ adaptations during TF-CBT, which is a valuable addition to the research field. The overall results suggest that the implementation of TF-CBT is feasible without a tremendous amount of adaptation. In line with other studies on cultural adaptations to trauma-focused treatments, therapists also made changes in the conceptualization of the trauma’s effects such as spiritual approaches (Ennis et al., 2020), and tailored materials and language (e.g. usage of metaphors) to the target group.

The additional content or techniques described were mostly TF-CBT components. Consequently, therapists had to repeat some components in later sessions (like psychoeducation before starting the trauma narrative) or brought components forward that were supposed to be carried out later (like affective modulation in the first session to enable some self-efficacy in dealing with PTSD symptoms). This is an approach that is typical for TF-CBT, which is meant to be flexible in the use of its components (Cohen et al., 2017). It is not surprising that dealing with the ‘crisis of the week’ was the content added most often. In addition to their trauma history, URMs have to deal with daily stressors and post-migration stressors (Keles et al., 2018) such as an unsecure asylum status, discrimination, or language and cultural barriers in the acculturation process. Therefore, we recommend that enhanced problem management related to post-migration stressors should be added as an additional component in TF-CBT (‘crisis of the week’) for this population.

There were reports of obstacles related to TF-CBT in only one third of cases. Language was named as the only problem in treatment, the other responses referred to components of little help for the respective treatment case. Relaxation was named most often. We recommend, however, to retain this content as part of the treatment as it is considered to be an important part of stabilization ahead of the trauma confrontation and suitable for use across different cultures.

The cultural adaptations named by the therapists were rather diverse. Looking at the data, we can see that most adaptations named were techniques that we would use irregularly throughout treatment, like the meaning of pride, strength or respect that can be included in the cognitive work, trauma narrative or future safety. The use of pride or respect and culture-specific grief rituals could be discussed in the TF-CBT training in order to enable therapists to deliver culturally sensitive treatment for URMs. This specific content might not be necessary for all URMs in treatment though, which means that therapists need to evaluate the inclusion of such culture-specific rituals and concepts for each individual patient independently. Therapists need to be trained to maintain a balance between cultural considerations and an overestimation of cultural aspects as this might lower manual adherence. In addition, suitable metaphors should be provided for different modules.

Even though trauma confrontation with asylum seekers is discussed in a controversial manner in the literature (ter Heide et al., 2016), the therapists named the trauma narrative as the most helpful component for their patients. The factor named most helpful for therapists was “having an agenda” which is not a TF-CBT component, but a characteristic of manualized CBT approaches. The agenda for every session seems especially helpful when the “crisis of the week” is a very dominant part of the treatment sessions.

There are several limitations which might limit the generalizability of the findings. Unfortunately, Question 3 about cultural adaptations was phrased as a closed, two-stepped question (“Did you consider cultural factors in this treatment case? If so, which ones?”). This might have forced a “yes” or “no” answer and might therefore have biased the results. Furthermore, we were not able to calculate interrater reliability scores for the coding of categories. In addition, there was a lack of objective ratings (e.g. independent raters of videos from treatment sessions). Lastly, we did not assess the therapists’ cultural competence or prior experience in transcultural work. Therefore, we cannot rule out that the level of cultural knowledge influenced the actual cultural adaptations.

### Conclusion

The present study enhances our knowledge about the implementation of TF-CBT for a culturally diverse sample such as URMs in Germany. Nonetheless, URMs face numerous individual and structural barriers to receiving mental health care interventions tailored to their needs. Within the project “BETTER CARE – Improving mental health care for unaccompanied young refugees through a stepped-care approach” (Rosner et al., 2020) we will implement TF-CBT according to the knowledge gained from the present study. Furthermore, the recommendations discussed can contribute to the implementation of TF-CBT in culturally diverse groups in future research and clinical practice and might help practitioners to overcome barriers in treating young refugees.

## Supplementary Materials

The Supplementary Material contains examples from the coding manual for the questions regarding additional techniques, obstacles to treatment and cultural adaptations (for access see Index of Supplementary Materials below).

10.23668/psycharchives.5029 Supplement 1 Supplementary materials to "“Same same or adapted?” Therapists’ feedback on the implementation of trauma-focused cognitive behavioral therapy with unaccompanied young refugees"

UnterhitzenbergerJ.
HaberstumpfS.
RosnerR.
PfeifferE.
 (2021). Supplementary materials to "“Same same or adapted?” Therapists’ feedback on the implementation of trauma-focused cognitive behavioral therapy with unaccompanied young refugees"
[Additional information]. PsychOpen. 10.23668/psycharchives.5029
PMC967082936405672
