# Supplementary material for: “Same Same or Adapted?” Therapists’ Feedback on the Implementation of Trauma-Focused Cognitive Behavioral Therapy With Unaccompanied Young Refugees
Source: Clin Psychol Eur. 2021 Nov 23;3(Spec Issue):e5431. doi: 10.32872/cpe.5431 (PMC9670829; doi:10.32872/cpe.5431)
Supplement: Supplement 1 [file cpe-03-5431-s01.pdf]

Supplementary material

“Same same or adapted?” Therapists’ feedback on the implementation of trauma-focused cognitive behavioral therapy with unaccompanied young refugees

Unterhitzenberger, J., Haberstumpf, S., Rosner, R., & Pfeiffer, E.

Manuscript published in Clinical Psychology in Europe

https://doi.org/10.32872/cpe.5431

Table A

Examples from the Coding Manual on Additional Techniques

Category	Content	Example
Crisis of the week	Mention of conflicts arising from the flight situation or the asylum process, discussion of recent incidents, recent problems (e.g. in school, training), current mood	"Patient is in conflict between right to care for himself and his family's hardships in his home country" (T 2)
Psychoeducation	Mention of any psychoeducational content on TF-CBT components, psychotherapy, on PTSD or comorbid symptoms, or on interventional techniques regarding those outside of the regular psychoeducation module	"Psychoeducation on fight-flight-freeze reactions" (T 4)
Cognitive processing	Repetition of cognitive processing component or mention of any techniques that involve working with cognitions outside of the regular cognitive processing module	"Description of thought stopping technique" (T 6)
Trauma narrative	Repetition of trauma narrative component or mention of any content or techniques that involve the trauma narrative or working with it outside of the regular cognitive processing module	"Identification of the worst moment" (T 2)
Other additional content	Mention of comprehensive content or techniques, feedback on therapy,	"Talk on if and how the patient would recommend therapy"

Note. PTSD = Posttraumatic stress disorder; *TF-CBT* = Trauma-focused cognitive behavioral therapy; *T* = Therapist

Table B

Examples from the Coding Manual on Obstacles Regarding TF-CBT

Category	Content	Example
Relaxation	Mention of relaxation component or any technique that involves relaxation	"The patient boycotted relaxation techniques" (T 6)
TF-CBT components ahead of trauma narrative	Mention of any content that describes period of time until trauma confrontation as too long	"Long duration of treatment until the trauma narrative: this enhanced avoidance in the patient and I would have preferred to do without relaxation and affective modulation" (T 4)
Linguistic problems	Mention of any linguistic problems	"Cognitive restructuring was almost impossible because of linguistic problems – despite the presence of an interpreter" (T 6)

Note. TF-CBT = Trauma-focused cognitive behavioral therapy; *T* = Therapist

Table C

Examples from the Coding Manual for Cultural Considerations

Category	Content	Example
Pride	Mention of pride from the patient's perspective	"The concept of lost pride due to sexual violence was new to me and needed discussion." (T 1)
Religion	Mention of any content involving religion or faith	"Religious rituals as a resource" (T 8)
Metaphors and proverbs	Mention of any metaphors or proverbs supplied by the patient	"Metaphors and proverbs that are now established in my work" (T 7)
Mental and somatic complaints	Mention of any attitudes, coping or experiences with mental and/or somatic complaints	"Attitudes towards mental illness and coping with somatic complaints" (T 6)

Note. T = Therapist
